# HIV PrEP Coverage Among Black Adults: A Concept Analysis of the Inequities, Disparities, and Implications

**DOI:** 10.1089/heq.2023.0250

**Published:** 2024-05-15

**Authors:** Ikenna Obasi Odii, David E. Vance, Patricia A. Patrician, Tracey K. Dick, Jenni Wise, Jessica L. Corcoran, Latesha Elopre, Crystal Chapman Lambert

**Affiliations:** ^1^ School of Nursing, University of Alabama at Birmingham, Birmingham, Alabama, USA.; ^2^ Division of Infectious Disease, University of Alabama at Birmingham, Birmingham, Alabama, USA.

**Keywords:** HIV/AIDS, HIV prevention, pre-exposure prophylaxis, Black adults, HIV PrEP coverage, health inequities, health disparities

## Abstract

**Background:**

Significant racial disparities exist in HIV pre-exposure prophylaxis (PrEP) coverage in the United States (U.S), with Black individuals experiencing seven times higher new HIV infection rates compared to their White counterparts. Despite being the highest priority population at risk for HIV, Black adults have the lowest PrEP coverage, impacting the overall progress toward meeting the ending the HIV epidemic (EHE) goals in the U.S.

**Methods:**

Utilizing the Walker and Avant method, this concept analysis examined existing literature and U.S. Centers for Disease Control and Prevention resources to explore HIV PrEP coverage.

**Results:**

Findings highlighted a lack of clarity in the concept, resulting in four operational definitions. To address this ambiguity, a conceptual definition of HIV PrEP coverage was proposed, focusing on equitable access to PrEP medication among sexually active individuals aged 18–64 years, particularly those traditionally underserved and would benefit from PrEP. This inclusive definition aims to align with the dynamics of sexual behavior in racial minority groups. Key attributes of this conceptual definition include estimates of PrEP use, access, need, cost, side effects, frequency of HIV testing, and self-efficacy. Antecedents entail HIV status, testing behaviors, transmission risks, and communication with health care providers. Consequences involve perceptions of risk, screening routines, provider biases, stigma, and potential HIV transmission reduction.

**Conclusion:**

Analyzing HIV PrEP coverage offers useful insights into social and structural factors exacerbating health inequities in the field of HIV prevention and control. This concept analysis underscores the importance of unified sexual health communication, diverse approaches to PrEP access for racial minorities, and improved sexual health policies for Black adults. Moreover, understanding and advocating for equity in HIV PrEP coverage is crucial for addressing the existing racial disparities and achieving the EHE objectives in the U.S.

## Introduction

Prevention represents the best approach to the ending of the HIV disease burden. A key prevention strategy is pre-exposure prophylaxis (PrEP), a novel medication used to protect HIV-negative people from HIV infection.^1^ PrEP offers a biomedical strategy for HIV prevention or transmission, and evidence suggests that optimal adherence to PrEP lowers the odds of contracting HIV by 99%.^1^ Being one of the six HIV indicators to monitor progress for ending the HIV epidemic (EHE), PrEP is an integral strategy for fighting the HIV epidemic.^2^ Yet, significant barriers, such as medical mistrust, stigma, personal biases of medical staff, and health care avoidance, continue to impede the awareness and utilization of HIV PrEP among Black adults.^3^ In this article, Black adults refers to people aged 18–64 years having origins in any of the Black racial groups of Africa. The article references Black adults in the same way that the racial distribution of HIV PrEP data is characterized all through the empirical literature.

Black adults account for 42% of all new HIV infections despite comprising 13% of the total U.S. population.^4–6^ Given the potential public health impact of PrEP on the HIV epidemic, concerns abound regarding the extent of uptake of PrEP among racial minority groups at substantial risk for HIV infection, which may eventually define the overall success of the EHE goal of the U.S. government. Despite being effective, significant racial disparities exist in HIV PrEP coverage in the United States, as well as contradictions regarding the meaning of HIV PrEP coverage, leading to questions such as: (a) What is HIV PrEP coverage? and (b) What are the racial disparities in HIV PrEP coverage? Perhaps, the disparity in HIV PrEP coverage in Black adults may currently be exacerbated by insufficient literature to both highlight the problem and clarify “HIV PrEP coverage” as a concept.

An examination of the HIV PrEP coverage statistics in the past four years (2019–2022) indicates successive years of both underutilization and the lowest coverage for Black Americans compared with other racial demographics. Unlike White people, who had an average PrEP coverage of 74%, and Hispanics/Latino people with an average of 18.9%, Black people had an average of 10.3% PrEP coverage (comparatively the lowest by race) in the past four years.^7^ Overall, PrEP coverage statistics expressed in percentage for Blacks were 8% in 2019, 9.2% in 2020, 11% in 2021, and 12.8% in 2022 (see Table 1). Although some minuscule progress is shown in the uptake of PrEP, PrEP coverage is still grossly disproportional, particularly for the number of Black persons eligible for PrEP. Thus, Blacks have the least coverage of HIV PrEP and are the least likely to meet the U.S. target goal of 50% PrEP coverage by 2025 and 90% PrEP coverage by 2030, thereby contributing to existing health disparities.^8^

**Table 1.** HIV PrEP Coverage by Race in the United States (%)

Race	2019	2020	2021	2022
African Americans/Blacks	8	9.2	11	12.8
Hispanics/Latino	14.5	16.4	20.4	24.4
Whites	60	64	78	93.8
Others	9.3	10	12	14.6

Health equity is attained when all people realize their highest level of health possible, apparent in equal valuing of everyone.^9^ The demonstrable inequity and racial disparity in HIV PrEP coverage among Black adults are exacerbated by structurally negated social determinants of health or significant socioeconomic barriers (e.g., income inequality, low educational background) limiting overall health care access and adherence to antiretroviral treatment for HIV.^10^ Hence, Black adults currently lag behind in taking steps toward meeting the EHE target goals, and all indications point to a significant lack of awareness, interest, uptake, or familiarity with HIV PrEP.^11^ The recently updated HIV PrEP initiation guidelines recommend education for PrEP to anyone who is sexually active and permit a PrEP prescription in the absence of sexual behaviors.^12,13^ During clinical visits, some patients may not be willing to disclose their extent of HIV transmission risk behaviors, which underscores the need to explore newer ways of increasing access and utilization of HIV PrEP, particularly for Black Americans.^14–17^ Examining current HIV-related disparities and health inequities regarding HIV PrEP coverage would contribute significantly to highlighting the overall HIV disease burden among Black adults.

Furthermore, current evidence indicates that the concept of HIV PrEP coverage is routinely contradictory in the literature, contributing to conflicting definitions or significant ambiguity in its understanding and interpretation.^18–21^ Apart from the rendition of the Centers for Disease Prevention and Control, HIV researchers have defined HIV PrEP coverage unilaterally with significant contradictions. For example, while some researchers refer to HIV PrEP coverage in terms of PrEP-to-need ratio, others associate it with PrEP use-to-HIV diagnosis ratio, or PrEP prescription-to-PrEP eligibility ratio.^18,19,22^ These renditions are further clarified in the results section of this article. Furthermore, some researchers opine that HIV PrEP coverage means the clients’ HIV negative status compared with PrEP enrollment, or PrEP eligibility of clients when compared with PrEP adherence.^21,23^ Therefore, lack of clarity and misunderstanding of “HIV PrEP coverage” as a concept is clearly evident in the existing literature. Specific changes to the conceptual definition of HIV PrEP coverage may both highlight the public health inequities disproportionately affecting Black adults and portend racial implications for the control of new HIV infections in this demographic.

The purpose of this concept analysis was to examine the existing literature, identify gaps in HIV PrEP knowledge, explore the inconsistencies in identified operational definitions of HIV PrEP coverage, and better conceptually redefine this concept with the aim of promoting consistency in using it for practice, research, and sexual health communication involving Black adults. From this, a synthesis of the findings was provided to articulate implications for health equity that could propel the science.

## Methods

### Walker and Avant Method

In this article, the Walker and Avant method of concept analysis was adopted to assess the concept of HIV PrEP coverage among Black adults in the U.S.^24^ HIV PrEP medication debuted in 2012, but uptake has been slow in the general population.^18^ We acknowledge the availability of several precursor articles prior to 2012. However, the focus of this concept analysis is on HIV PrEP coverage as from 2012 when it received official approval from the Food and Drug Administration (FDA).

The current guidelines for PrEP eligibility that was revised in December 2021 by the U.S. Centers for Disease Control and Prevention (CDC) were relied upon during the conceptualization and completion of this article. Walker and Avant’s method is appropriate for this concept analysis because HIV PrEP coverage requires clarification to understand its attributes, antecedents, and consequences in practice and research. This method is also suitable because HIV PrEP coverage as a concept is unlikely to be altered conceptually by the dynamics of one individual’s sexual behavior. Developing a clear conceptual definition will be helpful in underpinning its operational (measurable) definition. The Walker and Avant method is an eight-step method composed of the following steps: (1) selecting a concept, (2) determining the aim of analysis, (3) identifying all uses of the concept, (4) defining attributes, (5) constructing a model case, (6) identifying antecedents and consequences, and (7) defining empirical referents.^24^ In addition to examining several uses of a concept, the Walker and Avant method identifies relevant components of the concept. This is particularly applicable when a concept is relatively new and may be a subject of ambiguity.

### Literature Search Strategy

The article search terms were: “HIV OR antiretroviral OR anti-HIV AND PrEP coverage, OR PrEP coverage, AND Blacks OR African American”. The search was narrowed using subject terms closely related to HIV PrEP coverage, such as “HIV PrEP coverage OR Anti-retroviral coverage”. HIV PrEP coverage was the main concept guiding the abstract and title review process. For a full review of selected articles, the following eligibility criteria were applied: 1) the study being conducted or published in the U.S. from 2012 to 2023; 2) published in the English language; 3) HIV PrEP coverage being discussed in the title or abstract; 4) was a primary source article. Full-text articles were then included in the review based on these inclusion criteria. For exclusion criteria, all studies conducted outside the U.S. were excluded, and articles with HIV but without HIV PrEP coverage or with HIV PrEP but without HIV PrEP coverage were excluded.

**FIG. 1.**
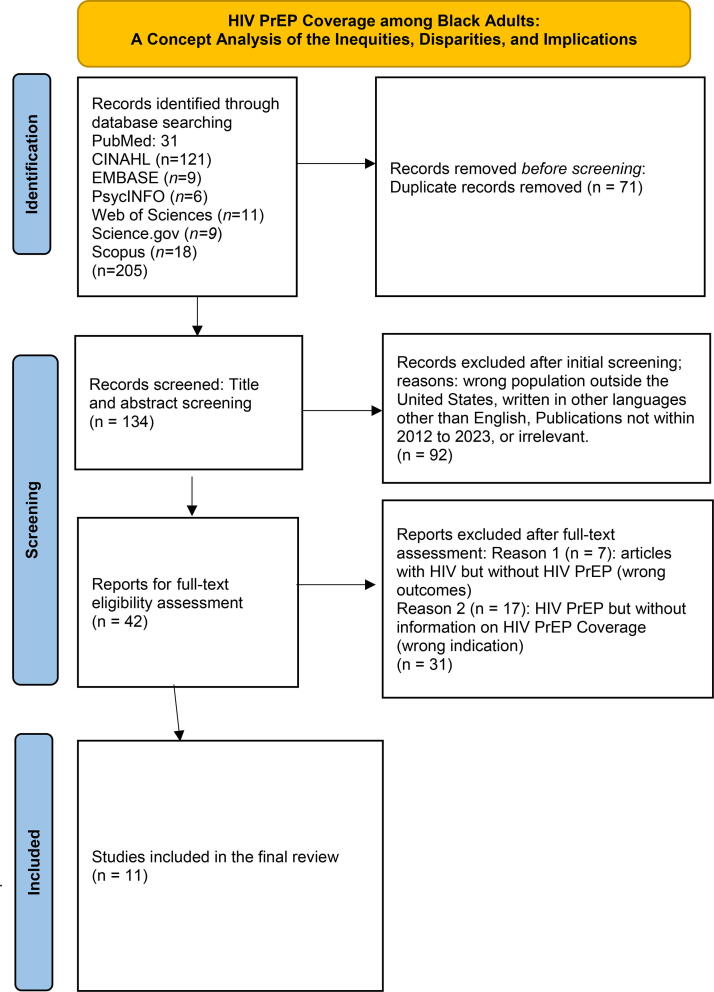
Literature review flow diagram.

As shown in Figure 1, a comprehensive exploratory search of 7 databases was conducted and resulted in: 31 articles from PubMed, 121 articles from CINAHL (EBSCOhost), 9 articles from EmBase, 6 articles from PsychInfo (ProQuest), 11 articles from Web of Science, 9 articles from Science.gov, and 18 articles from Scopus. Subsequently, the article search were refined to include studies conducted in the U.S. in English language. Publications from 2012 to 2023 were selected because the intervention protocol for PrEP was approved in 2012.

Initially, 205 articles were imported from the databases and saved in an EndNote library by one of the authors. Subsequently, the articles were exported into Covidence software, where 71 duplicate articles were removed, leaving a total of 134 articles that were screened for inclusion. At this juncture, only the titles and abstracts were screened. Ninety-two articles were considered irrelevant based on exclusion criteria, leaving 42 articles for full-text assessment. Following full-text assessment, 31 articles were excluded for lack of evidence and relevance. Finally, 11 articles met all inclusion criteria and were synthesized for the concept analysis.

## Results

### Operational Definitions of HIV PrEP Coverage

Operational definitions identify how concepts are measured and applied.^24^ In the context of any given year for adults, PrEP coverage is the number of people who have received a PrEP prescription divided by the overall number of individuals estimated to be eligible for PrEP and could benefit from the medication.^18^

Four significant renditions of HIV PrEP coverage were identified during the synthesis of operational definitions from 11 articles selected for inclusion in this review (see Table 2). However, two common themes were: HIV-negative people at risk for HIV infection or have an indication for PrEP and the number of people using PrEP or people who received a prescription for PrEP. HIV PrEP coverage was defined as a PrEP-to-need ratio and measured as the number of people using PrEP divided by the number of people newly diagnosed with HIV.^19,20,25,27,28^ All references to “need” refer to the need for HIV prevention as reflected in the use of PrEP, while the indication for PrEP pertains to HIV-negative individuals that may benefit from PrEP but have not attained HIV PrEP coverage. Similarly, HIV PrEP coverage was defined as the number of persons receiving a PrEP prescription divided by the number of persons estimated to be eligible for PrEP or had indications for PrEP.^18,22,26,27^ Conversely, Fojo et al.^23^ posit that HIV PrEP coverage implies HIV-negative individuals at risk for HIV infection but enrolled in a PrEP program. In contrast, Hamilton et al.^21^ advance the view that HIV PrEP coverage is the proportion of adults eligible for PrEP and currently using or adhering to the prescribed PrEP regimen.

**Table 2.** Summary of Empirical Referents of HIV PrEP Coverage with Operational Definitions

Ratio of No. of people using PrEP to No. of people diagnosed with HIV	Ratio of No. of people receiving PrEP prescription to No. of people with PrEP eligibility	HIV negative people at risk for HIV infection enrolled in a PrEP program	Proportion of adults meeting PrEP eligibility criteria using and adhering with PrEP regimen
Goedel et al. (2020)^25^	Centers for Disease Prevention and Control. (2021)^18^	Fojo et al. (2021)^23^	Hamilton et al. (2018)^21^
Patel et al. (2022)^19^	Harris et al. (2019)^26^		
Rosenberg & Marcus (2018)^27^	Smith et al. (2020)^22^		
Schexnayder et al. (2022)^20^	Rosenberg & Marcus (2018)^27^		
Sullivan et al. (2020)^28^			

### Defining Attributes of HIV PrEP Coverage among Black Adults

Attributes are the features or characteristics that are most often associated with a concept and that would make up the model case, thereby setting it apart from other similar concepts.^24^ Two key attributes in the literature that define HIV PrEP coverage are estimates of PrEP use (numerator) and estimates of PrEP need (denominator).^19,27^ Furthermore, these defining attributes are applicable to Black adults as follows: (1) the number of Black people with an indication for PrEP (estimates of PrEP need); (2) groups at risk for HIV transmission such as youths aged 18–34 years within the Black adult population (estimates of PrEP need); (3) Blacks within a given period of time, with demonstrable slower PrEP uptake compared with Hispanics and Whites (estimates of PrEP use); and (4) populations with lower levels of PrEP use relative to need such as Black adults in the United States and older Black adults [(estimates of PrEP use) see Table 3].

### Antecedents of HIV PrEP Coverage

Generally, HIV testing and sexual health programs precede eligibility for PrEP coverage because maintaining a HIV-negative status is a cardinal criterion for coverage to be initiated.^29^ Also, condomless anal sex is associated with a higher likelihood of HIV acquisition, particularly with partners whose HIV status are unknown.^30,31^ Chan et al.^32^ suggests that people with indications for PrEP in the United States alone may be up to 1.1 million. Similarly, the prevalence of certain sexual behaviors—previous or existing relationships among partners with unknown HIV status, injection drug use, multiple sex partnerships with unequal power dynamics, routine engagement in casual sex, a positive diagnosis of sexually transmitted infections (STIs)—are related antecedents of HIV PrEP coverage. Other antecedents include communication between patients and willing providers of health care regarding HIV prevention, adolescents and young adults at the age of sexual experimentation or early sexual debut, the ability to identify those in need of PrEP, and the ability of individuals to self-identify a need for PrEP (see Table 3).

**Table 3.** Summary of What Was Added in This Concept Analysis (Attributes, Antecedents, and Consequences)

Attributes	Antecedents	Consequences
Estimates of PrEP use and access (numerator) Estimates of PrEP need (denominator) Cost Side-effects Frequency of HIV testing Frequency at HIV setting Location of HIV care setting, Self-efficacy	Current HIV negative status HIV testing and sexual health programs HIV transmission risk behaviors Communication between patients and willing providers of health care regarding HIV prevention Density of PrEP clinics Young adults at the age of sexual experimentation or early sexual debut.	Routine HIV screening Potential of reduction in new HIV transmissions Risk reduction counseling Perception of self-perceived HIV risk PrEP stigma and discrimination in the context of race or sexual orientation Care provider related bias to PrEP

### Consequences of HIV PrEP Coverage

Walker and Avant conceptualized consequences as defining outcomes as a result of the concept.^24^ The consequences of PrEP coverage are discussed first as general consequences of PrEP coverage, and secondly as consequences of low PrEP coverage among Black adults.

#### General consequences of PrEP coverage

Apart from the desirable outcome of controlling or ending new HIV infections, interventions such as routine HIV and STIs screening are some of the consequences involved in maintaining PrEP coverage. Routine screening helps to ensure that providers are not prescribing PrEP to someone who seroconverts and serves as standard protocols in PrEP coverage. Routine screening is also a valuable way of reducing HIV diagnosis rates and facilitating PrEP adherence counseling, especially in the highest priority populations such as Black adults.^31,33^ However, the overall impact of screenings on HIV reduction remains inconclusive in the literature,^33^ despite the acclaimed gains of PrEP use such as reduction in new HIV transmissions and increased linkage to HIV care. Other consequences of PrEP coverage include the need for sexual health and harm reduction counseling targeted at consistent use of condoms during sexual intercourse, behavioral therapy for substance abuse if addiction is indicated, and consistent use of sterile equipment for injection drug users (see Table 3).

#### Consequences of low PrEP coverage among Black adults

Black adults are the highest priority demographic that ought to benefit from PrEP coverage to ameliorate the widening health disparity evident in the literature. Low PrEP coverage increases the risk for contracting HIV, and the consequences are evident in the form of worsening individual and structural barriers such as sex work, self-perceived low HIV risk, PrEP stigma, and discrimination in the context of race or sexual orientation.^21,29^ Provider-related barriers such as lack of familiarity with PrEP guidelines or protocols, confidentiality challenges, lack of health insurance coverage, cost, and distance accessing PrEP clinics were some of the consequences of low PrEP coverage. All of these factors erode the efforts being made to reduce the risk of new HIV diagnoses among Black adults,^29^ eventually diminishing opportunities to identify PrEP candidates, improve linkages to PrEP care, and lower barriers to PrEP adherence (see Table 3).

### Examining a Model Case

For this concept analysis, the following model case is presented.

Brandon, a cisgender 24-year-old Black male, secures his routine appointment with the nurse practitioner (NP) with complaints of minor sprain in his ankle sustained during a soccer bout. On general examination, Brandon is assessed to be healthy with no history of cardiovascular or endocrine disease, bacterial sexually transmitted infections in the past 6 months, and no history of substance abuse. However, following the clinical visit, Brandon requested to speak in confidence with his NP, and he is obliged. He informs the NP that he wants to start taking HIV PrEP and that he had read about the efficacy of the HIV prevention medication. When the NP asks for his reasons for requesting for PrEP, he simply says he needs the PrEP medication without disclosing any HIV risk factors. The NP is unsure why Brandon requests PrEP and probes further to rule out recent possible HIV exposure. In the past, Brandon had not reported having any sexual contact with anyone and denies any sexual affiliation with his female companion at school. Eventually, the NP refers Brandon to the physician who decides to implement a same-day PrEP initiation protocol: conducting an HIV test and assessment of renal functions to ensure that he is HIV-negative and meets the criteria for safely initiating PrEP. Brandon eventually receives a prescription of oral Truvada, and is offered PrEP literacy counseling to ensure he clearly understands the requirements of adherence and follow-up visits. Moreso, he is placed on a medication assistance program and scheduled for a 3-monthly routine clinical visit in the interim.

From the model case, Brandon was clearly not open to disclosing his sexual risk behavior and so does not showcase indications for PrEP in sexually active persons. Instead, he verbalizes a need for PrEP, which is consistent with empirical findings of general patient aversion to the disclosure of levels of sexual risk behavior in HIV care.^14–17^ His knowledge of PrEP coverage may have led to quicker access to PrEP, coupled with his belonging to the highest priority demographic experiencing the most new HIV infection burden and yet enduring the least PrEP coverage. Moreover, the defining attributes of PrEP coverage, such as the need for PrEP and the ability to access it, are fully reflected in the model case. Similarly, cost, portrayed by the medication assistance program—a defining attribute of an acceptable and culturally sensitive PrEP program that promotes PrEP coverage—is also evident in the model case. Brandon expresses a need for PrEP in the absence of specific sexual behaviors, and he is provided a prescription after counseling to enhance PrEP adherence, HIV, renal, and STI screenings. This scenario conforms with the new CDC guideline, which mandates providers to make PrEP available to patients with either a high risk for HIV infection, sexually active adult patients irrespective of sexual orientation and gender, or people who request for PrEP in the absence of specific sexual behaviors.^14,15^ Furthermore, the newly articulated conceptual definition of HIV PrEP coverage aims to promote HIV disease prevention among Black adults while advocating for inclusive PrEP access among racial minority groups such as Black adults. These specific aims are evident in the model case.

### Empirical Referents of HIV PrEP Coverage

This concept analysis article on HIV PrEP coverage is, to the best of our knowledge, a novel article primarily targeting Black adults. As far as we know, there is no concept analysis article exclusively on HIV PrEP coverage in the literature. Hence, referents in the literature are articles that provided definitions of HIV PrEP coverage either exclusively or inadvertently, while addressing other similar or contrasting populations, outcomes, or indications. Tables 2 and 3 summarizes the 11 articles that provided empirical referents for the concept of HIV PrEP coverage.

## Discussion

HIV PrEP coverage is suboptimal among Black adults in the United States compared with other racial demographics such as Whites and Hispanics, which is likely driven by multiple determinants. The primary purpose of this article was to examine the existing literature regarding the uses of HIV PrEP coverage, while aligning our findings in highlighting inequities in access to HIV PrEP among Black adults and the racial disparities in the domestic HIV burden. Overall, we discovered the existence of four different definitions of the concept in 11 empirical literatures that met the inclusion criteria. Regarding what is newly added by this article, several key issues related to the concept, such as cost, location of HIV care setting, and other relevant social determinants of health, were rigorously scrutinized and summarized together as either attributes, antecedents, or consequences of the concept. Outlining a more inclusive conceptual definition and analyzing several related facets of HIV PrEP coverage might be a useful first step in both highlighting the problem, clarifying the concept, and eventually addressing the inequity.

### Health Equity Implications for Black Adults in the United States

From this concept analysis, there are several health equity implications for Black adults in the United States. First, considering that four conflicting operational definitions of PrEP coverage were discovered across the literature, HIV PrEP coverage requires a more inclusive conceptual definition that highlights a need for racial equity. At the moment, empirical referents indicate the existence of multiple contradictory definitions of what constitutes HIV PrEP coverage (see Table 2). A more robust definition of HIV PrEP coverage should integrate the U.S. goal of EHE while accounting for the existing racial disparities in PrEP coverage. Given the reasons why Black adults experience the highest rate of new HIV infection and the lowest HIV PrEP coverage compared with the general population, a fitting conceptual definition may be a helpful step in driving stakeholder engagement and optimizing the participation of Black communities in PrEP programs. Individual and structural factors [i.e., poverty, lack of access to health care (uninsured or under-insured), lack of awareness of HIV status, higher rates of some STIs, & HIV stigma] have also long been associated with the racial disparities among Black adults^5,10,34–37^ A conceptual definition of PrEP coverage should reflect these factors, in addition to the attributes of this concept earlier identified. Hence, we conceptually redefine HIV PrEP coverage as *equitable PrEP coverage determined by the number of filled PrEP medication prescriptions among sexually active persons aged 18–64 years who would benefit from PrEP or traditionally experience lower levels of PrEP use*. This conceptualization includes all the defining characteristics of HIV PrEP coverage and appropriately captures PrEP use to need ratio. It affords future researchers a better conceptualization of what PrEP coverage entails, and the impact of access to PrEP for all who need them, as one of the most effective tools for HIV prevention.

Second, considering the antecedents and attributes of HIV PrEP coverage identified in this article such as cost and location, HIV prevention efforts should primarily focus on supporting existing efforts at integrating PrEP programs into other comprehensive public health services for Black adults and streamlining existing fragmented system of PrEP access. This will further support the under-insured and uninsured in the Black community to navigate several initiatives currently riddled with varying eligibility and rules. Moreover, compared with 76% of the U.S domestic HIV/AIDS budget that is spent on HIV treatment and care, HIV prevention is poorly funded and receives only 3% of the federal domestic HIV/AIDS budget.^38^ Increasing HIV prevention budget to fund newer and existing Black HIV prevention community-led programs will ameliorate the current challenges regarding cost, self-efficacy, location of HIV prevention care setting, or perception of side effects and unfavorable perception about PrEP, which are attributable to the concept (see Table 3). Sustaining recent progress in HIV prevention will require advocacy for HIV PrEP coverage equity as a useful tool for the overall HIV epidemic control in the United States. The high HIV burden among Black adults should be a source of concern that prompts the reallocation of PrEP resources and programs to Black communities and health centers. One prevalent operational definition of HIV PrEP coverage in the literature is the PrEP-to-need ratio, which evaluates whether the demand for HIV prevention is adequately met by current PrEP usage. In 2021 alone, Black adults accounted for 42% of new HIV diagnosis but experienced only 14% of PrEP use.^39^ This low ratio is clearly indicative of unmet need for PrEP coverage. Comparatively, Whites accounted for 26% of new HIV diagnosis but experienced 65% PrEP use.^39^ The existence of glaring HIV PrEP coverage inequity should serve as the basis for articulating policies and programs aimed at significantly increasing Black adults’ linkage to PrEP care. This article further contributes to the existing literature in highlighting the potentials of having HIV prevention programs that match the realities of Black adults who may benefit from them.

Third, given that cost, confidentiality, acceptability, location, and frequency of care setting are routinely highlighted as attribute concerns for accessing PrEP, further interventions for Black adults are warranted. The CDC alludes to these social determinants of health as key drivers of HIV PrEP coverage inequities (i.e., social and economic marginalization), which makes it harder to achieve HIV prevention for Black adults.^7,40^ Therefore, the formation and linkage of Black social peer networks comprising clients accessing PrEP who consent to participate may be a functional option to support Black adults in overcoming access or adherence to care and the aforementioned issues. Black social peer networks should be supported with no-cost-to-patient PrEP resources for consumers who have made at least one clinical visit. New consumers who may feel more comfortable accessing PrEP through peers in the Black community should be offered an alternative pathway to access it. Evidence shows that a public health approach that situates HIV PrEP services closer to everyone who needs them will be critical in achieving HIV epidemic control in the United States.^41^ Similarly, health care practitioners should prioritize universal PrEP counseling, while organizations focusing on HIV prevention and control should consider investing more in HIV PrEP commercials during Black social and community events. For Black communities where social peer networks may be impossible to initiate, health care providers at the local level should be mandatorily trained to be knowledgeable about and achieve comfort in prescribing PrEP. Given that PrEP messaging may be misconstrued as a sole intervention for sexual and gender minority groups, efforts should also be focused on commercials highlighting PrEP use in heterosexual Black adults and heterosexual sero-discordant Black couples. This re-orientation will contribute to enhancing comprehensive knowledge and acceptability of PrEP in Black communities.

Lastly, future interventions on HIV PrEP should focus on building evidence for newer generations of HIV prevention products that provide options for potential consumers, such as long-acting injectable PrEP or flexible silicone vaginal ring. Also, further studies should aim at testing empirical indicators of PrEP coverage, such as sexual health communication and structural factors like the medication assistance program for uninsured and under-insured consumers, in order to evaluate and address their impact on HIV prevention. Furthermore, emphasis should be placed on engaging Black adults during the development of HIV prevention interventions to promote service utilization and cultural congruity for Black adults.

### Limitations and Strengths

This concept analysis did not consider some Whites who may share similar indications for PrEP or existing social determinants of health with Black people. Also, this concept in other sociocultural contexts with contrasting social and policy frameworks may be fundamentally different than that identified in this analysis. Although four conflicting operational definitions of PrEP coverage were discovered across the literature and a conceptual definition was proposed, there may be a possibility of missed resources in gray literature or unscreened databases. Findings from this concept analysis article may not be generalizable beyond contexts with similar U.S. public health care systems, given that articles for inclusion in this review were restricted to publications focused on the U.S. Furthermore, the measurement criteria for PrEP coverage in operational definitions of this concept may be difficult to understand or delineate without expert clarification. Conversely, this article is perhaps the first review article analyzing HIV PrEP coverage and situating the concept within the realities of Black adults in the U.S. Moreover, this article passed several technical and methodological rigor while applying some of the most scrupulous software available for conducting comprehensive searches for systematic themed reviews.

## Conclusion

Equity in HIV PrEP coverage is paramount for HIV prevention, coupled with potential public health implications for Black adults. Indeed, health inequities and racial disparities currently exist in HIV PrEP coverage within the United States. This concept analysis identified four conflicting operational definitions of HIV PrEP coverage in the literature: the ratio of the number of people using PrEP to the number of people diagnosed with HIV, the ratio of the number of people receiving PrEP prescription to the number of people with PrEP eligibility, HIV-negative people at risk for HIV infection enrolled in a PrEP program, and the proportion of adults meeting PrEP eligibility criteria adhering to a PrEP regimen. Furthermore, this article outlines new information on attributes, antecedents, consequences, and a proposed definition of HIV PrEP coverage, which focuses on inclusivity and equitable PrEP access by Black adults. Analyzing HIV PrEP coverage among Black adults could be an integral first step toward advocacy for increasing access to HIV prevention methods that work best for Black adults and fit into their lifestyle. To turn the tide on HIV transmission among Black adults, there is a need to formally integrate PrEP counseling into all comprehensive health programs, improve supply chain planning for all PrEP options, streamline fragmented systems of PrEP access, enhance HIV PrEP delivery in Black neighborhoods, and invest in a new generation of HIV prevention products.

## Abbreviations Used

CDC: Centers for Disease Control
EHE: Ending the HIV Epidemic
FDA: Food and Drug Administration
HIV: Human Immuno-Deficiency Virus
NP: Nurse Practitioner
PrEP: Pre- Exposure Prophylaxis
STIs: Sexually Transmitted Infections
U.S: United States
